# Immunoregulatory functions of innate lymphoid cells

**DOI:** 10.1186/s40425-018-0433-8

**Published:** 2018-11-19

**Authors:** Sarah Q. Crome, Pamela S. Ohashi

**Affiliations:** 1 Toronto General Hospital Research Institute, University Health Network, Toronto, ON M5G 1L7 Canada; 2 Department of Immunology, Faculty of Medicine, University of Toronto, Toronto, ON Canada; 3 Princess Margaret Cancer Centre, University Health Network, Toronto, ON M5G2M9 Canada; 4 Department of Medical Biophysics, University of Toronto, Toronto, ON Canada

**Keywords:** Innate lymphoid cells, Natural killer cells, T cells, Regulation, Myeloid derived suppressor cells, Regulatory T cells, Inflammation, Cancer, Regulatory ILCs, Immune homeostasis

## Abstract

Innate lymphoid cells (ILCs) are increasingly being recognized for their ability to impact both innate and adaptive immune cells in diverse contexts. ILCs have been observed in all secondary lymphoid tissues, in addition to being tissue-resident innate lymphocytes. In these locations, ILCs are poised to interact with various immune cells at different stages of an immune response. While the heterogeneity and plasticity of ILCs has complicated their study, their association with immune dysregulation in a wide range of pathologies highlights their importance to human health and disease. Notably, in addition to promoting inflammatory immune responses, populations of ILCs have been shown to inhibit immune responses through a variety of mechanisms. The reports of ILC-mediated regulation of immune responses have differed in terms of the phenotype of the regulatory ILC populations, and their mechanism of action. Yet the ability to modulate immune responses appears to be an important function of ILCs. As our understanding of this family of lymphocytes evolves, delineating the factors that dictate whether ILCs orchestrate inflammatory immune responses or suppresses these responses will be important for understanding various disease mechanisms. Here we focus on recent reports that examine how ILCs regulate immunity in different contexts.

## Commentary

Innate lymphoid cells (ILCs) are a recently described family of lymphocytes that have important functions in inflammation, host defence and tissue remodelling. Unlike B and T cells, these lymphocytes do not possess antigen specific receptors, but rather rapidly respond to environmental signals from tissues and other immune cells. Natural Killer (NK) cells, the founding member of the ILC family exhibit cytotoxic functions analogous to CD8^+^ T cells. Other ILC family members are not cytotoxic and have been categorized based on the conservation of transcription factors and cytokines that parallel CD4^+^ T helper (Th) cells [1]. Due to the ability to produce IFN-γ and the requirement for T-bet expression for development and function, cytotoxic NK cells as well as non-cytotoxic ILC1s are characterized as Group 1 ILCs. Group 2 ILCs produce IL-4, IL-5, IL-9 and/or IL-13 and require Gata-3 and RORα for their development. Group 3 ILCs produce IL-22 alone or in combination with IL-17 and utilize RORγt for their development. While these definitions have provided a helpful framework to study ILCs, they do not adequately describe the complexities of this family of lymphocytes. ILCs exhibit substantial plasticity and display variable effector functions that are closely linked to the tissue microenvironment [2]. For example, TGF-β in the tumor microenvironment was recently shown to induce mouse NK cells to convert to non-cytotoxic ILC1s that fail to control tumor growth or metastasis [3, 4]. Complicating the study of ILCs further is the large degree of heterogeneity, even within a particular subset. For instance, between 3,500 and 30,000 phenotypically separable subsets of NK cells were observed within peripheral blood of healthy individuals [5]. These factors, in addition to phenotypic and functional differences between mouse and human ILCs, has hampered the ability to establish a consensus definition of ILC subsets. Despite these challenges, the ability of ILCs to influence immune responses has led to tremendous interest in understanding their role in a wide range of pathologies.

ILCs have many protective functions, yet populations of have ILCs have also been associated with chronic inflammation and autoimmune diseases including asthma, inflammatory bowel disease, graft-versus-host disease (GVHD), psoriasis, rheumatoid arthritis (RA) and atopic dermatitis [6]. Regulation of ILC differentiation, activation and function are therefore important for the outcome of an immune response. Intriguingly, in addition to promoting T cell responses, ILCs have been shown to inhibit inflammatory immune responses and mouse studies have demonstrated the potential to directly limit T cell responses in transplantation, autoimmunity, and host defence [7, 8]. Antibody depletion of NK1.1^+^ ILCs in mice prior to infection with the lymphocytic choriomeningitis virus prevented establishment of chronic infection [9, 10], as NK1.1^+^ ILCs in these studies, were killing helper and cytotoxic T cells. ILC2s and ILC3s have also been linked with immunosuppressive activity. Both are capable of processing and presenting model antigens in vitro, and MHC II-dependent interactions in the absence of co-stimulation resulted in ILC3-mediated inhibition of CD4^+^ T cell responses to intestinal commensal bacteria in mice [8, 11]. Several recent studies have highlighted the importance of ILC regulation in a broad range of immune contexts. While the ILC populations described vary, these reports demonstrate that ILCs may act similar to regulatory T cells (Tregs) and inhibit both innate and adaptive immune cells.

ILCs promote inflammation in a variety of rheumatic diseases [6]. While much work has focused on understanding the signals that lead to pathological inflammation, identification of immune cells and cytokines required to resolve inflammation are less well defined. A recent study by Rauber et al. described an essential role for IL-9 producing mouse ILC2’s in initiating the resolution of inflammation in RA [12]. Based on the observation that antigen-induced arthritis did not spontaneously resolve in *Il9*^−/−^ mice, the authors investigated whether an IL-9 producing population was critical for the resolution process. While overexpression of IL-9 did not impact the initiation phase of RA, it strongly accelerated the resolution of inflammation. Of the cytokines that remained persistently high in the absence of IL-9, IL-17 was the only cytokine that differed between WT and *Il9*^−/−^ mice, and an increase in CD4^+^ T helper 17 (Th17) cells was observed in the joints of *Il9*^−/−^ mice. This aberrant Th17-mediated immune response was due to poor Treg suppressive function and occurred in the absence of an IL-9-producing ILC population that promoted Treg suppressive abilities. Due to expression of ST2, ICOS, CD25, CD90 and Sca1, these IL-9 producing ILCs were defined as an ILC2, and direct cell contact through GITR-GITRL and ICOS-ICOSL interactions mediated their interactions with Tregs. These mouse studies were supported by an observation of reduced ILC2s in the synovium of RA patients. Furthermore, ILC2 numbers in patient blood correlated with disease activity, as there was a significant reduction in ILC2s in patients with persistent inflammation but significantly higher numbers in patients in remission. Collectively, these studies support that ILC2s may be important for limiting Th17-mediated inflammation through promotion of Treg activity.

ILC1s and ILC3s have been linked with the maintenance of mucosal integrity, promotion of lymphoid organogenesis and orchestration of immune responses to intestinal pathogens and commensal bacteria. ILC1s undergo expansion in human Crohn’s disease, and when dysregulated, ILC3s promote ulcerative colitis. In a recent study by Wang et al., a distinct ILC population was identified that suppressed the activation of ILC1s and ILC3s via secretion of IL-10, leading to protection against innate-driven intestinal inflammation [13]. TGF-ß1 was secreted by these regulatory ILCs and acted in an autocrine manner to maintain this population. When the authors compared the gene expression profile of these regulatory ILCs to Tregs and other ILCs, they did not express the Treg-transcription factor Foxp3 nor exhibit a transcription factor profile consistent with other ILC family members. Rather, ID3 (inhibitor of DNA binding 3) was shown to be the critical transcription factor controlling their development. The authors proposed that this distinct ILC population serves as a counterbalance to pro-inflammatory ILC1 and ILC3s, and instead favors the resolution of intestinal inflammation. While the ability of an ILC population to control innate intestinal inflammation is intriguing, studies addressing consequences of regulatory ILCs on intestinal T cell responses will be important, as dysregulated T cell responses occur in Crohn’s disease and ulcerative colitis. It would also be interesting to compare the ILCreg gene expression profile to T regulatory 1 cells (Tr1) cells, the IL-10 secreting Treg population that does not express Foxp3 and is an important regulator of intestinal inflammation. Notably, production of IL-10 may be a common characteristic of immunosuppressive ILCs, as previous reports identified IL-10 producing NK cells with regulatory activity [7, 14], and Seehus et al. recently observed an ILC2 population that secreted IL-10 and was associated reduced lung inflammation in mice [15]. IL-10 producing ILC2s were expanded in the lung with in vivo IL-2 administration, and while they contracted after the cessation of stimulation, a subset was maintained that could be recalled upon restimulation. This suggests IL-10 producing ILC2s could serve a tissue-resident regulatory innate immune population to rapidly control local inflammation.

ILC-mediated suppression of immune responses may also impact anti-tumor immunity. While investigating the potential of using tumor-infiltrating lymphocytes (TIL) as an adoptive T cell-therapy for high grade serous cancer (HGSC), we identified a regulatory CD56^+^ ILC population capable of directly regulating T cells [16]. These regulatory CD56^+^ ILCs inhibited T cell expansion and altered CD4^+^ and CD8^+^ TIL cytokine production. Remarkably, the presence of regulatory CD56^+^ ILCs within a patients TIL was associated with a striking reduction in time to recurrence in HGSC patients. Regulatory CD56^+^ ILCs expressed the natural cytotoxicity receptors (NCRs), NKG2/CD94 family members and killer immunoglobulin receptors (KIRs), and were CD56^bright^, which are all expressed by NK cells. Yet they shared phenotypic properties with other ILCs, as they produced IL-22, and in some patients IL-9, *GATA3*, *AHR* and *RORΑ* transcription factors in addition to *TBET* and *EOMES,* and exhibited low cytotoxicity to conventional NK target cells, K562 cells. Of note, non-suppressive tumor-infiltrating CD56^bright^ ILCs were distinguished from regulatory CD56^+^ ILCs by a different gene expression profile and high production of IFNγ and TNFα in the absence of IL-22. While CD56^bright^ NK cells have been previously described as regulatory, both for their potent cytokine production, and also for their ability to directly suppress T cells [7, 14], many of these studies were performed prior to the identification of ILC family members. Our findings suggest tissue-resident CD56^bright^ ILCs include innate lymphocytes with distinct functions, thus it will be important to re-examine the CD56^bright^ ILCs in these contexts, and determine whether these regulatory ILCs are a result of distinct developmental pathway, or are conventional NK cells that have been altered by the microenvironment. NKp46 interactions regulated suppression of expansion, as addition of anti-NKp46 antibodies enhanced T cell expansion. Many questions remain, however, in terms of cellular interactions that control CD56^+^ regulatory ILC crosstalk with T cells, the contexts in which this occurs, and the role of NKp46 in regulating this process.

CD56^bright^ NK cells and activated ILC2 and ILC3s have been associated with decreased incidence of GVHD following hematopoietic stem cell transplantation (HSCT) [7, 17]. A recent study by Bruce et al. raised the possibility that ILCs could be used as a cell-based therapy to prevent GVHD [18]. Using a mouse model of allogenic BMT, ILC2s in the lower GI tract were shown to be particularly sensitive to conditioning therapy and displayed a limited ability to repopulate from donor bone marrow. ILC2 infusion was associated with increased myeloid derived suppressor cells (MDSCs) and reduced donor Th1 and Th17 cells. Expansion of MDSCs was dependent on ILC2 production of IL-13 and corresponded with improved intestinal barrier function. Notably, protection from GVHD was not associated with inhibition of the graft-versus-leukemia (GVL) effect. While the authors did not directly examine ILC-T cell interactions, ILC promotion of MDSCs indirectly inhibited pathogenic T cell responses. This study raises the possibility that ILC2s could be targeted or used as a cell-based therapy to promote tolerance with HSCT. However, inhibiting ILC2s may be beneficial in the context of cancer, as highlighted by findings by Trabanelli et al. [19] In this study, tumor-derived PGD2 and B7-H6 activated and expanded ILC2s in the context of acute promyelocytic leukaemia. These activated ILC2s promoted monocytic myeloid-derived suppressor cell (M-MDSC) activation through IL-13 secretion and were associated with poorer tumor control. In a humanized mouse model, blocking PGD2, IL-13 or NKp30 partially restored ILC2 and M-MDSC levels, and corresponded to increased survival. Thus, ILC2s may limit anti-tumor immune responses indirectly via modulation of myeloid cell function.

The mechanisms behind ILC-mediated regulation are only beginning to be understood, but emerging evidence supports that in addition to indirect regulation via cytokine production or effects on antigen presenting cells or other ILCs, direct ILC-T cell interactions shape the outcome of different immune responses. Regulatory mechanisms observed include regulation of T cells via killing, antigen-presentation in the absence of co-stimulation, expression of inhibitory molecules, suppressing T cells via IL-10 or adenosine and inhibition of memory T cell formation [7, 8]. While ILCs are tissue-resident sentinels, they have also been described within all secondary lymphoid tissues [8], central sites for activation of T cells and B cells. In these various locations, ILCs may serve as key regulators controlling immune responses at different stages. The multitude of regulatory mechanisms may therefore reflect a requirement to control responses in different spatial and temporal contexts. A greater understanding of ILCs regulatory capabilities, how they arise and are controlled, and how they exert their immunosuppressive functions is therefore necessary to define the regulatory role of ILCs in human health and disease.

## Abbreviations

GVHD: Graft-versus-host disease
HSCT: Hematopoietic stem cell transplantation
IFN: Interferon
IL: Interleukin
ILC: Innate lymphoid cell
MDSCs: Myeloid derived suppressor cells
NK cell: Natural killer cell
NKp46: Natural cytotoxicity receptor
RA: Rheumatoid arthritis
TGF-β: Transforming growth factor beta
Th17 cell: CD4+ T helper cell
TNF: Tumor necrosis factor

## References

[1] Spits H (2013). Innate lymphoid cells-a proposal for uniform nomenclature. Nat Rev Immunol.

[2] Spitz H, Bernink JH, Lanier LL (2016). NK cells and type 1 innate lymphoid cells: partners in host defense. Nat Immunol.

[3] Gao Y (2017). Tumor immunoevasion by the conversion of effector NK cells into type 1 innate lymphoid cells. Nat Immunol.

[4] Cortez VS (2017). SMAD4 impedes the conversion of NK cells into ILC1-like cells by curtailing non-canonical TGF-β signaling. Nat Immunol.

[5] Horowitz A (2013). Genetic and environmental determinants of human NK cell diversity revealed by mass cytometry. Sci Transl Med.

[6] Shikhagaie MM (2017). Innate lymphoid cells in autoimmunity: emerging regulators in rheumatic diseases. Nat Rev Rheumatol.

[7] Crome SQ (2013). Natural killer cells regulate diverse T cell responses. Trends Immunol.

[8] Withers David R. (2016). Innate lymphoid cell regulation of adaptive immunity. Immunology.

[9] Waggoner SN (2012). Natural killer cells act as rheostats modulating antiviral T cells. Nature.

[10] Lang PA (2012). Natural killer cell activation enhances immune pathology and promotes chronic infection by limiting CD8^+^ T cell immunity. Proc Natl Acad Sci U S A.

[11] Hepworth MR (2015). Group 3 innate lymphoid cells mediate intestinal selection of commensal bacteria-specific CD4^+^ T cells. Science.

[12] Rauber S (2017). Resolution of inflammation by interleukin-9-producing type 2 innate lymphoid cells. Nat Med.

[13] Wang S (2017). Regulatory innate lymphoid cells control intestinal inflammation. Cell.

[14] Poli Aurélie, Michel Tatiana, Patil Neha, Zimmer Jacques (2018). Revisiting the Functional Impact of NK Cells. Trends in Immunology.

[15] Seehus CR, et al. Alternative activation generates IL-10 producing type 2 innate lymphoid cells. Nat Commun. 2017:1900. 10.1038/s41467-017-02023-z. 10.1038/s41467-017-02023-z PMC5711851 29196657

[16] Crome SQ (2017). A distinct innate lymphoid cell population regulates tumor-associated T cells. Nat Med.

[17] Vacca P (2016). NK cells and other innate lymphoid cells in hematopoietic stem cell transplantation. Front Immunol.

[18] Bruce DW (2017). Type 2 innate lymphoid cells treat and prevent acute gastrointestinal graft-versus-host disease. J Clin Invest.

[19] Trabanelli S (2017). Tumour-derived PGD2 and NKp30-B7H6 engagement drives an immunosuppressive ILC2-MDSC axis. Nat Comm.

